# Toward Highly Thermal Stable Perovskite Solar Cells by Rational Design of Interfacial Layer

**DOI:** 10.1016/j.isci.2019.11.007

**Published:** 2019-11-09

**Authors:** Weitao Yang, Danming Zhong, Minmin Shi, Shaoxing Qu, Hongzheng Chen

**Affiliations:** 1 State Key Laboratory of Silicon Materials, MOE Key Laboratory of Macromolecular Synthesis and Functionalization, Department of Polymer Science and Engineering, Zhejiang University, Hangzhou 310027, P.R. China; 2 State Key Laboratory of Fluid Power & Mechatronic System, Key Laboratory of Soft Machines and Smart Devices of Zhejiang Province, Department of Engineering Mechanics, and Center for X-Mechanics, Zhejiang University, Hangzhou 310027, P.R. China

**Keywords:** Energy Storage, Materials Characterization Techniques, Energy Materials, Devices

## Abstract

Heat is crucial to the long-term stability of perovskite solar cells (PVSCs). Herein, thermal stability of PVSCs based on metal oxide (MO) and polymer (P) was investigated. Firstly, chemical decomposition behavior of perovskite films was characterized and analyzed, revealing that chemically active MO would accelerate the decomposition of methylamine lead iodide (MAPbI_3_). Secondly, thermal-induced stress, resulting from the mismatched thermal expansion coefficients of different layers of PVSCs, and its effect on the mechanical stability of perovskite films were studied. Combining experiment and simulation, we conclude that “soft” (low modulus) and thick (>20 nm) interfacial layers offer better relaxation of thermal-induced stress. As a result, PVSCs employing thick polymer interfacial layer offer a remarkably improved thermal stability. This work offers not only the degradation insight of perovskite films on different substrates but also the path toward highly thermal stable PVSCs by rational design of interfacial layers.

## Highlights

• Degradation of perovskite films on different substrates were studied
• The chemical and mechanical deterioration of perovskite films are distinguished
• Thermal-induced stress within PVSCs was eased by a soft and thick interfacial layer
• PVSCs with optimized interfacial layers showed superior thermal stability

## Graphical Abstract

**Figure:**
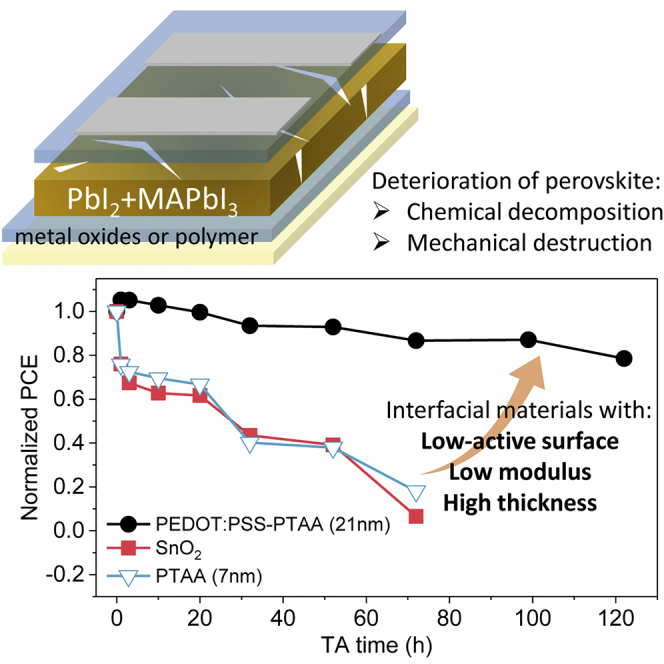

## Introduction

Perovskite solar cells (PVSCs) based on halide lead perovskite such as methylamine lead iodide (MAPbI_3_) have emerged as one of the most promising candidates for solar energy harvest due to their outstanding photo-electronic properties and compatibility to large-area solution-processing fabrication. Nowadays, the power conversion efficiency (PCE) of the state-of-the-art PVSCs has been over 23% with small-area, and over 15% with large-area (Jiang et al., 2019a, Jung et al., 2019, Deng et al., 2018, Li et al., 2018). Instability of PVSCs, therefore, becomes the major bottleneck for their commercial applications. In the last decade, degradation mechanism of perovskite absorber as well as the over-roll device has been widely studied. Heat, illumination, moisture, and oxygen are considered to be the four most essential environmental factors that are responsible for deterioration of PVSCs (Boyd et al., 2019, Deretzis et al., 2018, Askar et al., 2017). Generally, the degradation of PVSCs is triggered by moisture and oxygen and would be accelerated under illumination or high temperature conditions (Askar et al., 2017, Bi et al., 2016, Juarez-Perez et al., 2016). Among them, moisture and oxygen issues could be tackled by hermetic encapsulation, which would also greatly reduce the negative influence of illumination (Jiang et al., 2019b, Han et al., 2015). However, it is reported that photovoltaic modules operating in hot climate could reach temperature of 65°C or even higher, and high temperature processes (such as annealing of device layer and device encapsulation) are involved in PVSCs fabrication (Boyd et al., 2019, Tress et al., 2019). On one hand, chemical and crystal phase deterioration of perovskite films would occur under thermal stress. Methods including perovskite ions engineering, defects passivation, and device engineering have been fully developed to achieve PVSCs with better thermal stability (Jiang et al., 2019a, Turren-Cruz et al., 2018, Bian et al., 2018, Niu et al., 2018, Wang et al., 2019, Chen et al., 2018). On the other hand, thermal-induced built-in stress would form within the PVSCs due to the mismatched thermal expansion coefficients of different layers in device (Rolston et al., 2018a, Rolston et al., 2018b; Jacobsson et al., 2015). Huang and his co-workers have reported a strain-accelerated degradation of perovskite film under illumination because of the increased ion migration (Zhao et al., 2017). What is more, a low toughness of <0.16 MPa m^0.5^ (comparable to that of NaCl crystal, which is 0.15–0.26 MPa m^0.5^) of perovskite crystal has been reported (Rolston et al., 2016, Ramirez et al., 2018). As a result, sufficient stress would accumulate within the device when operating under extreme temperature and thus induce fracture. However, few attention has been paid on tackling such thermal-induced stress. Fabricating PVSCs based on polymer substrates such as polyethylene terephthalate (PET) instead of glass seems to be an alternative approach to reduce such built-in stress (Zhao et al., 2017, Hu et al., 2019). But their inferior moisture/oxygen blocking ability and relatively low hardness compared with their inorganic counterparts hold back their further applications. Hence, detailed influences of thermal aging on the PVSCs stability are worth further investigations, and strategies to improve the heat endurance of PVSCs need to be developed to meet the requirement of practical applications.

Herein, thermal stability of perovskite films based on substrates with various interfacial layers of metal oxides and polymers was investigated. Based on the results of X-ray diffraction, optical microscope, etc., the chemical and mechanical origins responsible for the degradation of perovskite films were distinguished. Further mechanics simulation and thermal stability test of full PVSC devices suggest that a thick and soft polymer interfacial layer with low-active surface is preferred for highly thermal stable PVSCs. This work elucidates the influence of interfacial layer properties on the thermal stability of PVSCs, providing a simple route to PVSCs capable for practical use by rational choice of interfacial layer materials.

## Results and Discussion

### Decomposition of MAPbI_3_ under Thermal Aging

**Figure 1:**
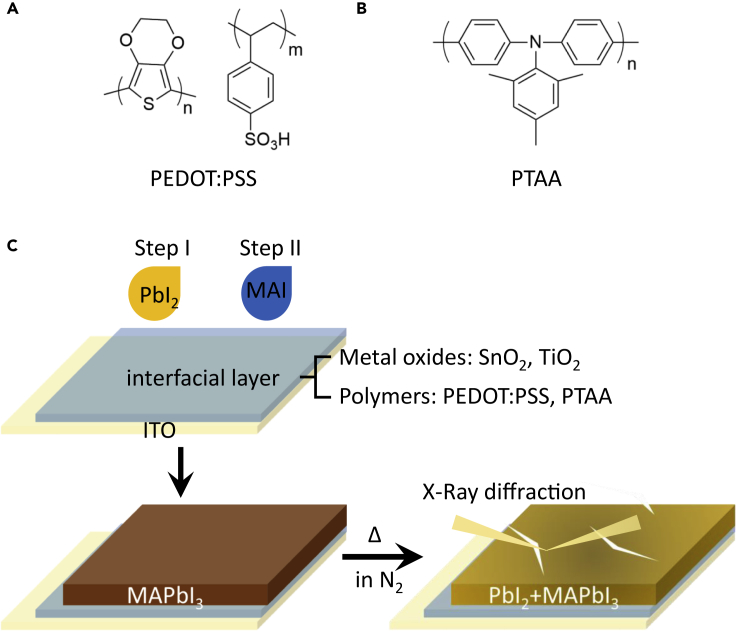
Molecular Structures and Sample Characterization (A and B) The molecular structures of PEDOT:PSS (A) and PTAA (B). (C) Diagram of MAPbI_3_ perovskite preparation on different interfacial layers and their thermal aging testing.

ITO glass substrates coated with four different interfacial layers of compact tin oxides (SnO_2_), compact titanium oxides (TiO_2_), poly(3,4-ethylenedioxythiophene):polystyrene sulfonate (PEDOT:PSS Al 4083), and poly[bis(4-phenyl) (2,4,6-trimethylphenyl)amine] (PTAA) were prepared according to the previous reports (Huang et al., 2016, Liu et al., 2018, Yang et al., 2019, Zhou et al., 2014). The molecular structures of PEDOT:PSS and PTAA are presented in Figures 1A and 1B, respectively. Tested by a stylus profiler, the thickness of SnO_2_, TiO_2_, PEDOT:PSS, and PTAA layers is determined to be 30 nm, 30 nm, 24 nm, and 7 nm, respectively. We choose these four materials not only because they belong to the two most widely used categories (metal oxide and polymer) of interfacial layer materials in PVSCs but also because they represent three mechanical property conditions including “tough and thick” (as SnO_2_, TiO_2_), “soft and thick” (as PEDOT:PSS), and “soft and thin” (as PTAA). These merits enable an ideal platform for us to carry out the study via well-designed trials. MAPbI_3_, one of the most commonly used and well-studied organic-inorganic hybrid perovskites for photovoltaic applications, was then deposited on different interfacial layers via a two-step method (Huang et al., 2016, Kojima et al., 2009, Chen et al., 2017). Details about the experimental conditions could be seen in the Supplemental Information. X-ray diffraction (XRD) patterns and top-view scanning electron microscope (SEM) images of different fresh perovskite films are shown in Figure S1. No significant difference in crystallinity and morphology is observed, indicating similar perovskite quality on different substrates. Finally, the as-prepared perovskite films were put on hotplate with various temperatures for thermal aging in glove box (see Figure 1C).

**Figure 2:**
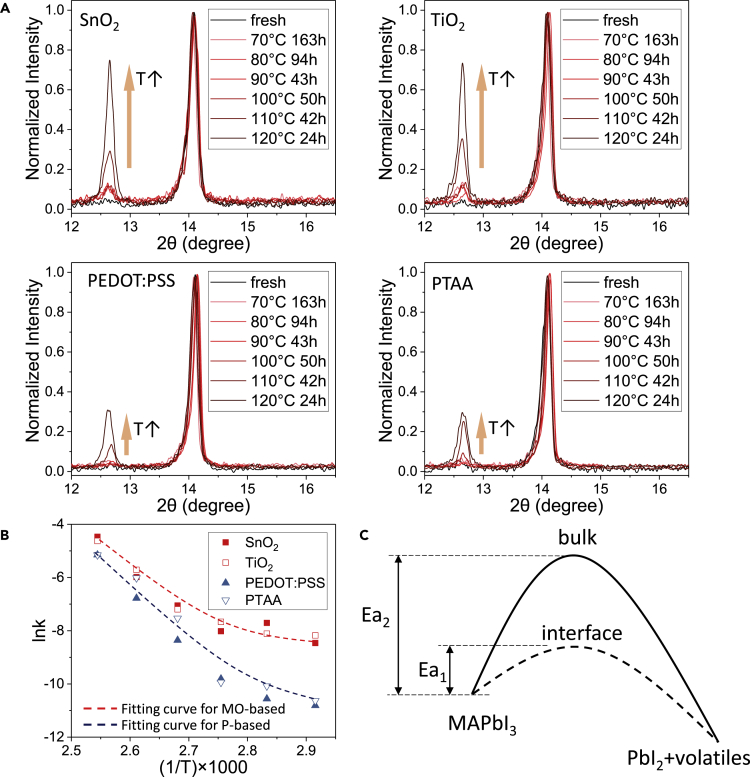
Chemical Deposition Analysis (A) XRD patterns of perovskite films prepared on different substrates after thermal aging on various conditions. (B) lnk-(1/T) plots of perovskite films on different interfacial layers. (C) Diagram of decomposition process taking place in bulk (solid line) and in interface (dash line). Also see Figures S1–S3.

X-ray diffraction characterization was performed to examine the decomposition of MAPbI_3_ films. As shown in Figure 2A, two peaks corresponding to PbI_2_ and MAPbI_3_, respectively, were detected at 12.7° and 14.1° (Zhao et al., 2017). By quantifying the peak area of PbI_2_ and MAPbI_3_ under various thermal stress, we could determine the decomposition rate (k) of MAPbI_3_ by:

$$\text{k}=\frac{S_{PbI_{2}}}{\left(S_{PbI_{2}}+b\cdot S_{MAPbI_{3}}\right)\Delta t}$$

where Δt is the thermal aging duration and S_PbI2_ and S_MAPbI3_ are the peak areas of PbI_2_ and MAPbI_3_, respectively. *b* = 1.92 is the calibration factor determined as the ratio of the intensity of PbI_2_ (001) plane signal in totally aged perovskite sample to the intensity of MAPbI_3_ (110) plane signal in fresh perovskite sample (see Figure S2). Background PbI_2_ signals have been removed by measuring the XRD spectra of the corresponding fresh perovskite films. Different k values under six different temperatures various from 343K to 393K were then measured and calculated, offering lnk-(1/T) curves of perovskite films deposited on different interfacial layers shown in Figure 2B. Depicted in Figure 2B, perovskite films deposited on metal-oxides-based (MO-based) layers (SnO_2_ and TiO_2_) suffer from higher decomposition rates than those deposited on polymer-based (P-based) layers (PEDOT:PSS and PTAA). What is more, high lnk tails at low temperature range were observed for MO-based perovskite films. Assuming that there are two parallel reactions during the decomposition process, we could fit the lnk-(1/T) data according to the Arrhenius equation:

$$\text{k}=A_{1}e^{-E_{a1}/RT}+A_{2}e^{-E_{a2}/RT}$$

where A and Ea are the pre-exponential factor and apparent activation energy for each decomposition process, R = 8.314 J/(mol∗K) is the gas constant, and T is the absolute temperature. Shown in Figure 2B and Table 1, an identical Ea_2_ (169.7 kJ/mol) for MO-based and P-based perovskites was observed at high temperature region, which could be related to the bulk-dominated decomposition of perovskite (Smecca et al., 2016, Yu et al., 2017, Deretzis et al., 2015). This value is also consistent with the previous reported values obtained by different methods (Smecca et al., 2016, Yu et al., 2017, Deretzis et al., 2015). More importantly, the A_1_ of MO-based perovskite (0.0595) is ∼6.8 folds higher than that of the P-based perovskite (0.0088), indicating significant enhancement in lower Ea (Ea_1_ = 16.6 kJ/mol) process at lower temperature region for MO-based perovskite. Considering the high k and low Ea features of this process, we suggest it should be related to the excess decomposition of perovskite catalyzed by residual active species on the MO/perovskite interface. To verify our hypothesis, isonicotinic acid (molecular structure shown in Figure S3 inset), a reported effective interfacial passivation molecule, was employed to prepare a self-assembling monolayer (SAM) on the MO-based substrates (Zuo et al., 2015, Zuo et al., 2017). Details about the preparation of SAM could be seen in the Supplemental Information. As a result, the k value significantly drop from −8.2 for reference substrates to −9.4 for SAM-passivated substrates under thermal stress at 363 K (shown in Figure S3), which is very close to that of the P-based substrates (lnk = −9.8). Based on these results, we argue that the chemically active MO-based substrates would offer extra interfacial decomposition pathway for perovskite (illustrated in Figure 2C). P-based interfacial materials with low-activity surface are preferred for PVSCs with better thermal stability.

**Table 1:** Parameters Used to Fit the Experimental lnk-(1/T) Data

Interfacial Layer	A_1_	E_a1_ (kJ/mol)	A_2_×10^−20^	E_a2_ (kJ/mol)	R^2^
MO-based	0.0595	20.8	3.64	169.7	0.9994
P-based	0.0088	20.8	2.06	169.7	0.9988

### Mechanical Fracture of MAPbI_3_ Film and Simulation

**Figure 3:**
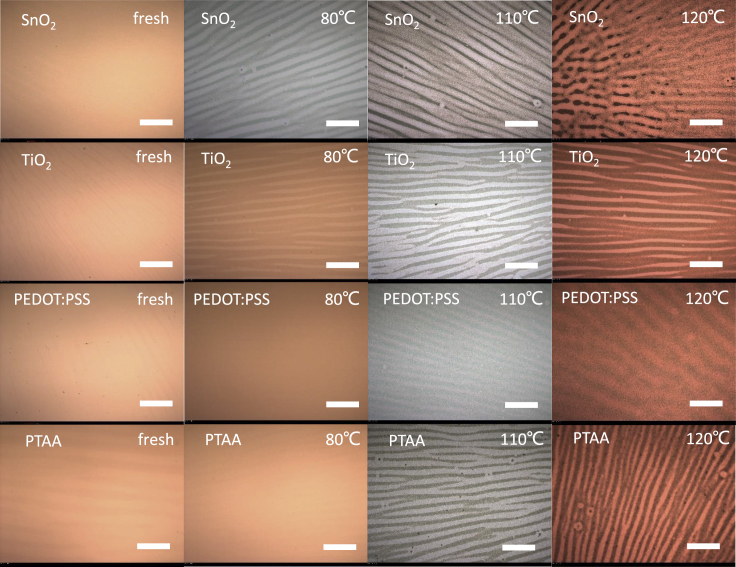
Optical Microscope Images of Perovskite Films Optical microscope images of perovskite films deposited on different interfacial layers under thermal aging with different temperatures. Scale bars in all pictures represent 200 μm.

The influence of thermal aging temperature on the mechanical stability of perovskite films was studied. Researchers have emphasized the impact of mismatched thermal expansion coefficients of different layers in device on the mechanical stability of PVSCs during thermal annealing (Boyd et al., 2019, Jacobsson et al., 2015, Ramirez et al., 2018). Particularly, the thermal expansion coefficient of MAPbI_3_ reaches 1.57×10^−4^ K^−1^, which is six times larger than those of indium tin oxide (ITO) and soda lime glass (Jacobsson et al., 2015, Fabini et al., 2016). Given the fairly low fracture energy (<1.5 J/m^2^) of perovskite due to its ionic salt-like structure, PVSCs might suffer from significant destruction under intense thermal stress (Rolston et al., 2018b). Herein, optical microscope was adopted to examine the morphology of perovskite films under various thermal aging conditions. As shown in Figure 3, obvious wrinkling fracture was observed in MO-based perovskite films after thermal annealing at over 80°C for 94 h. Similar destruction of perovskite films could also be seen in perovskite films based on PTAA layer. Considering different chemical decomposition behaviors for MO-based and PTAA-based perovskites discussed in the above section, we ascribe these wrinkles to the mechanical fracture of perovskite films, which is resulted from the thermal-induced compressive stress. It is worth pointing out that perovskite films deposited on PEDOT:PSS interfacial layer possess much less wrinkles even under high temperature over 110°C than that deposited on PTAA and MOs. Considering the thicker (or softer) layer of PEDOT:PSS than PTAA (or MOs), we hypothesize that thickness as well as modulus of the interfacial layer might be essential to the mechanical stability of perovskite film under thermal stress.

**Figure 4:**
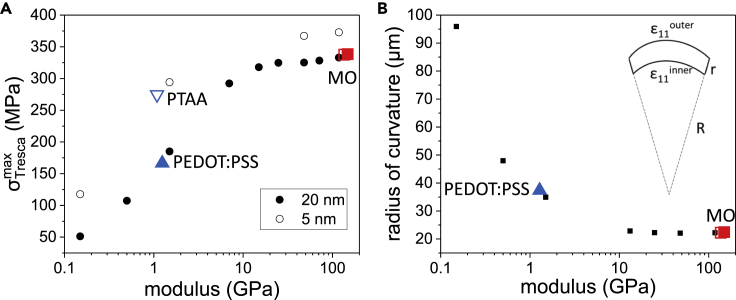
Mechanics Simulation (A) Dependence of max Tresca equivalent stress on the modulus and thickness of interfacial layer under a simulative temperature of 120°C. (B) Dependence of radius of curvature on the modulus of interfacial layer under a simulative temperature of 120°C. Simulative positions of the interfacial layers employed in this work are marked in the plots. Also see Figures S4–S6 and Table S1.

To have an in-depth understanding on the mechanical fracture of MAPbI_3_ films, we performed finite element simulation based on mechanical properties, and the conditions are summarized in Table S1. Tresca equivalent stress $\sigma_{Tresca}$ [defined as the maximal difference between principal stresses, i.e. $\sigma_{Tresca}=\text{max}\;\left(\left|\sigma_{1}-\sigma_{2}\right|,\;\left|\sigma_{1}-\sigma_{3}\right|,\;\left|\sigma_{2}-\sigma_{3}\right|\right)$, with the three principal stresses $\sigma_{1}$, $\sigma_{2}$, $\sigma_{3}$], which commonly serves as failure criterion for solid sample, is adopted to characterize the stress levels in the MAPbI_3_ layer. Herein, the dependence of maximum Tresca equivalent stress ($\sigma_{Tresca}^{\;max}$) within the perovskite layer on the modulus (E) and the thickness of interfacial layer was simulated. Exhibited in Figure 4A, the higher the modulus of the interfacial layer or the thinner the thickness of the interfacial layer, the higher the $\sigma_{Tresca}^{\;max}$ within the perovskite layer. Particular positions corresponding to SnO_2_, TiO_2_, PEDOT:PSS, and PTAA interfacial layer materials are marked in the plot, demonstrating considerably higher $\sigma_{Tresca}^{\;max}$ for MO-based ($\sigma_{Tresca}^{\;max}$> 300 MPa) and PTAA-based ($\sigma_{Tresca}^{\;max}$≈ 280 MPa) perovskite films than that of PEDOT:PSS-based perovskite film ($\sigma_{Tresca}^{\;max}$≈ 160 MPa). The higher stress level would lead to more serious destruction within the perovskite films, which is consistent with the optical microscope results in Figure 3. In addition, these results imply a threshold fracture stress of between 160 and 280 MPa. This value is consistent on the order of magnitude with the previous reported yield stress (∼740 MPa for single crystal perovskite), and tensile fracture stress (∼130 MPa for polycrystal perovskite film) of MAPbI_3_ perovskite, indicating good validity of this simulation (Reyes-Martinez et al., 2017, Ahn et al., 2019). In a word, the mechanics simulation suggests significant mechanical buffer effect of the thick and soft interfacial layer, providing reasonable explanation for the alleviated wrinkles of PEDOT:PSS-based perovskite film.

Secondly, due to the constraint effect of substrate on perovskite film, there would be unbalanced thermal expansion across the perovskite films (see Figure S4). Such distortion of perovskite films are transformed and represented by the equivalent radius of curvature (r) determined by r =L×(ε_11_^inner^+1)/(ε_11_^outer^-ε_11_^inner^), where L = 300 nm is the thickness of perovskite film and ε_11_^outer^ and ε_11_^inner^ are the ε_11_ (the strain along direction 1) adjacent to the surface and interface, respectively (illustrated in the right top inset of Figure 4B). The r-E curve was then depicted in Figure 4B, where a 2–5 folds larger r was obtained when E decreases from more than 10 GPa to 0.1–1 GPa, suggesting more intense distortion of perovskite films on tough interfacial layers than those on soft interfacial layers. Finally, mechanics simulation of the full device containing counter electrode (silver) and top interfacial layer ([6,6]-phenyl-C61-butyric acid methyl ester [PCBM]) was performed, demonstrating comparable results (illustrated in Figure S5). To further prove our simulation result, optical microscope images of perovskite films based on thick PTAA (∼30 nm) and thin PEDOT:PSS (∼8 nm) after thermal aging were collected. In spite of the low coverage of perovskite films (Figure S6) due to the bad wettability between PbI_2_ solution and PTAA surface, perovskite film based on thick PTAA shows much fewer wrinkling fracture than that on thin PEDOT:PSS after thermal annealing at 110°C (Figure S6). Based on these results, both experimental and simulative, we argue that a soft (with modulus of few GPa or less) and relatively thick (thickness >20 nm) interfacial layer would effectively release the thermal-induced stress within the PVSCs, contributing to improved thermal stability.

### Related to the PCE Degradation

**Figure 5:**
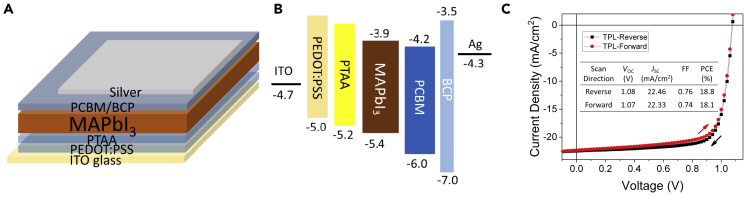
Device Structure, Energy Level Diagram, and PVSC Performance (A) Device structure of TPL-based PVSCs. (B) Energy level diagram of the PVSCs. (C) Forward (red) and reverse (black) scanned J-V curves of the champion TPL-based PVSC. Also see Tables S2 and S3.

PVSCs based on different interfacial layers were fabricated to evaluate the relationship between the thermal degradation of MAPbI_3_ film and the device performance. As shown in Table S2, the SnO_2_-, TiO_2_-, PEDOT:PSS-, and PTAA-based PVSCs show the maximum PCEs of 15.26%, 13.55%, 13.56%, and 17.15%, respectively, indicating good charge transporting capability of the above interfacial layers. In order to study the influence of the thickness of polymer interfacial layers on the thermal stability of the PVSCs, we further fabricated PVSCs with device structure of ITO/PEDOT:PSS/PTAA/MAPbI_3_/electron transport layer/Ag, where a bilayer thick polymer layer (TPL) was adopted. Device structure and energy level diagram of such TPL-based PVSCs was shown in Figures 5A and 5B. Optimization of TPL could be seen in Table S3. In brief, higher thickness leads to inferior charge transport capability, resulting in a decreased *J*_SC_, whereas too thin thickness leads to a low *V*_OC_. Finally, the optimized TPL possesses a thickness of ∼21 nm (with thickness of 14 nm and 7 nm for PEDOT:PSS and PTAA, respectively). Encouragingly, such TPL-based devices show a highest PCE of 18.8% (Table S2), probably due to the cascade energy level alignment (see Figure 5B) of ITO/PEDOT:PSS/PTAA/MAPbI_3_, which offer better charge collection efficiency. The J-V curve and parameters of the champion device was depicted in Figure 5C.

**Figure 6:**
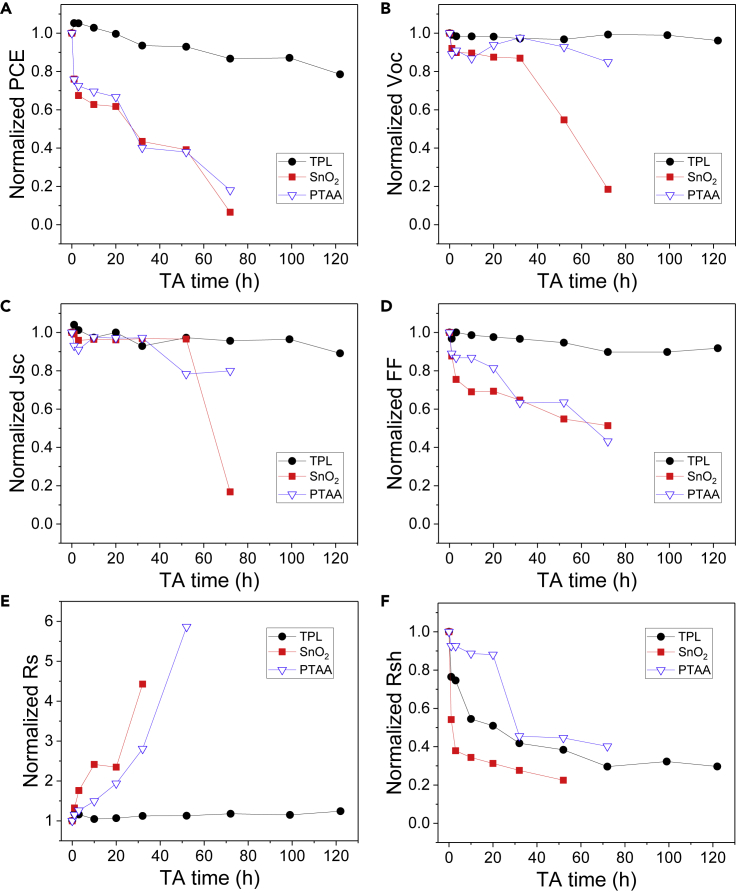
Device Thermal Stability Test (A–F) PCE (A), V_OC_ (B), J_SC_ (C), FF (D), Rs (E), and Rsh (F) evolution of PVSCs based on TPL (black solid circle), SnO_2_ (red solid square), and PTAA (blue hollow down triangle) interfacial layers heated at 100°C in glovebox. Also see Figure S7.

Then, PVSCs based on SnO_2_ (30 nm), PTAA (7 nm), and PEDOT:PSS/PTAA (21 nm) interfacial layers were fabricated, and their thermal stability were examined by placing different devices on 100°C hotplate in glove box. As shown in Figure 6A, the SnO_2_-based and PTAA-based PVSCs suffer from severe PCE deterioration in the first 24 h, remaining only 40% of their initial PCEs after thermal aging for 40 h. On the contrary, the TPL-based devices keep over 80% of their initial PCEs after over 120 h annealing. By replacing Ag with Au to eliminate the electrode corrosion, the thermal stability could be further improved, offering a T80 of more than 380 h (see Figure S7). Detailed parameters evolution (shown in Figures 6B–6D) indicates that fill factor (FF) decay is the primary factor responsible for the PCE drops. To understand the mechanism of such difference in device performance, sheet resistance (Rs) and shunt resistance (Rsh) evolutions were derived from the corresponding J-V curves and depicted in Figures 6E and 6F. On one hand, Rs of the SnO_2_-based and PTAA-based devices significantly increased and reached over five folds of their initial values, whereas Rs of the TPL-based PVSCs barely changed (see Figure 6E). On the other hand, greater decrease in Rsh was observed for SnO_2_-based PVSCs (see Figure 6F). According to the equivalent circuit description of photovoltaics, Rs is related to the bulk and contact resistance of the device, whereas Rsh is related to the leak current caused by defects or pinholes within the active layer (Wu et al., 2015). Hence, we reasonably attribute the increase in Rs and the decrease in Rsh to the fracture and decomposition of MAPbI_3_ layer according to our finding discussed in the former sections.

**Figure 7:**
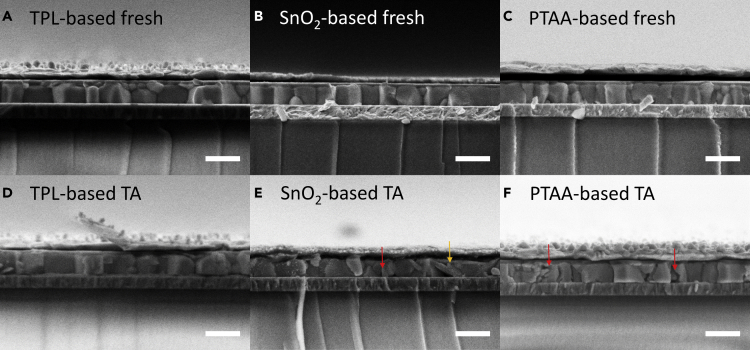
SEM Cross-Sectional Images of PVSCs (A–C) Cross-sectional images of TPL-based PVSC (A), SnO_2_-based PVSC (B), and PTAA-based PVSC (C) before thermal aging. Scale bars in all pictures represent 500 nm. (D–F) Cross-sectional images of TPL-based PVSC (D), SnO_2_-based PVSC (E), and PTAA-based PVSC (F) after thermal aging at 100°C. Scale bars in all pictures represent 500 nm.

We further verified our argument by conducting the cross-sectional SEM characterization. As shown in Figure 7B, the crystal fracture (marked by red arrow) features were observed in thermal-aged PVSCs based on both PTAA and SnO_2_ layers (Figures 7E and 7F), whereas obvious PbI_2_ product (flake-like feature marked by yellow arrow) could only be seen in SnO_2_-based PVSCs (Figure 7E). These results also agree well with the XRD characterization where much stronger PbI_2_ signal was observed in SnO_2_-based perovskite than in P-based perovskites after thermal aging at 100°C (Figures 1A–1C). In a word, the device performance test and SEM results further support our XRD and optical microscope characterizations and the corresponding conclusions. PEDOT:PSS/PTAA, a thick polymer interfacial layer, exhibits outstanding chemical and mechanical stability under thermal stress.

### Conclusion

In this work, influence of interfacial layer on the thermal stability of perovskite was thoroughly studied. The overall degradation of perovskite was distinguished into chemical decomposition and mechanical fracture via XRD and optical microscope characterizations, etc. Accelerated decomposition and worse mechanical destruction were observed in MO-based perovskite films, which are ascribed to the chemically active surface as well as high modulus feature of metal oxides interfacial layer materials. After conducting mechanics simulation and overall device stability test, we demonstrate the qualities desired for a good interfacial layer, including low modulus (few GPa or less), low chemical activity, and capability of thick layer (thickness >20 nm) utilization. In a word, our work offers not only the degradation insight of the perovskite films based on various substrates but also the path toward PVSCs with good heat endurance by rational design of interfacial layer materials.

### Limitations of the Study

In this study, a thick polymer interfacial layer is adopted to improve the high thermal stability of PVSCs. However, the deterioration of polymeric materials should be carefully considered under other aging conditions.

## Methods

All methods can be found in the accompanying Transparent Methods supplemental file.

## Supplemental Information

Document S1. Transparent Methods, Figures S1–S7, and Tables S1–S3
