# Supplementary material for: Toward Highly Thermal Stable Perovskite Solar Cells by Rational Design of Interfacial Layer
Source: iScience. 2019 Nov 9;22:534–43. doi: 10.1016/j.isci.2019.11.007 (PMC6920322; doi:10.1016/j.isci.2019.11.007)
Supplement: Document S1. Transparent Methods, Figures S1–S7, and Tables S1–S3 [file mmc1.pdf]

ISCI, Volume *22*

Supplemental Information

Toward Highly Thermal Stable Perovskite Solar Cells by Rational Design of Interfacial Layer

Weitao Yang, Danming Zhong, Minmin Shi, Shaoxing Qu, and Hongzheng Chen

Transparent Methods

Experimental Procedures

Materials: Dimethyl sulfoxide (DMSO), N,N-dimethylformamide (DMF), PbI_2 were purchased from TCI. Chlorobenzene (CB), ethanol, isopropanol (IPA), and toluene were bought from Sigma Aldrich. 15% SnO_2 colloid solution in water, 4-tert-butylpyridine (tBP), abis(trifluoromethylsulfonyl)amine lithium salt (Li-TFSI) and MoO_3 were purchased from Alfa Aesar. Bathocuproine (BCP), [6,6]-phenyl-C61-butyric acid methyl ester (PCBM), Poly[bis(4-phenyl)(2,4,6-trimethylphenyl)amine] (PTAA) were obtained from J&K., American Dye Source and Xi'an p-OLED, respectively. Methylammonium iodide (MAI) was purchased from Shanghai Mater Win New Materials. 4-Tert-butylpyridine (tBP) and bis(trifluoromethylsulfonyl)amine lithium salt (Li-TFSI) were purchased from Alfa Aesar. All materials were used without further purification. TiO_2 nanocrystal were systhesized based on the procedure reported by Yang Y. et al (Zhou et al., 2014).

Device Fabrication and Chracterization: Perovskite solar cells were fabricated on glass substrates pre-coated with a layer of indium tin oxides (ITO). The ITO substrates were cleaned sequentially by detergent, deionized water, acetone, and isopropanol for 15 min in an ultrasonic bath before fabrication. The pre-cleaned ITO substrates were then treated with UV-Ozone for 20 min. To deposit the SnO_2 layer, 15% SnO_2 colloid solution was diluted by deionized water to 5% and thereafter spun coat onto ITO substrates at 3500 revolutions per minute (rpm) for 45 s, followed by thermal annealing in air at 150 °C for 30 min. To deposit the TiO_2 layer, TiO_2 nanocrystal solution was spun coat onto ITO substrates at 3500 rpm for 45 s, followed by thermal annealing in air at 150 °C for 30 min. To deposit the PEDOT:PSS layer, PEDOT:PSS (Baytron P AI4083) was filtered through a polytetrafluoroethylene filter (0.45 μm) and thereafter spun coat onto ITO substrates at 3500 rpm for 45 s, followed by baking at 140 °C for 20 min. To deposit the thin PEDOT:PSS layer, PEDOT:PSS was diluted 4 times by deionized water and filtered through a polytetrafluoroethylene filter (0.45 μm), and then spun coat onto

ITO substrates at 3500 rpm for 45 s, followed by baking at 140 °C for 20 min to get a ~8 nm PEDOT:PSS thin film. To deposit the thin PTAA layer, the ITO substrates were transferred to a glovebox. Then a layer of PTAA (2 mg mL^{-1} in toluene) was spin-coated onto the cleaned ITO at 6000 rpm for 45 s. To deposit thick PTAA layer, a solution dissolved in 1 mL of toluene contained 15 mg of PTAA, 7.5 µL of LiTFSI stock solution (520 mg/mL in acetonitrile) and 7.5 µL of tBP. The PTAA solution was spun onto the perovskite film at 4000 rpm for 30 s to get a ~30 nm PTAA thick film. To deposit PEDOT:PSS/PTAA bilayer, PEDOT:PSS was diluted 2 times by deionized water and filtered through a polytetrafluoroethylene filter (0.45 µm), and then spun coat onto ITO substrates at 3500 rpm for 45 s, followed by baking at 140 °C for 20 min to get a ~15 nm PEDOT:PSS thin film. The pre-deposited substrates were then transferred to a glovebox and a layer of PTAA with thickness of 7 nm (2 mg mL^{-1} in toluene) was spin-coated onto them at 6000 rpm for 45 s.

Deposition of SAM follows previous literature (Zuo et al., 2017). In brief, isonicotinic acid was dissolved in methanol at a concentration of 0.5mg/mL, and was then spun onto the MO-based substrates at 3000 rpm. They were then annealed at 120°C for 15 min. Afterward, excessive isonicotinic acid was washed away by methanol.

PbI_2(DMSO) was dissolved in DMF at a concentration of 1 M. MAI was dissolved in IPA at a concentration of 50 mg mL^{-1}. To make the perovskite film, the PbI_2(DMSO) solution was spin coted onto the substrates with different interfacial layers at 3000 rpm for 30s, immediately followed by spin coating of MAI solution at 3000 rpm for another 30 s. Afterward, the obtained film were annealed at 90°C for 15 min. PCBM/BCP (or PTAA/MoO_3) charge transport layers were then deposited on the top of $MAPbI_3$ film to form structures of ITO/HTL/$MAPbI_3$/PCBM/BCP/Ag (or ITO/ETL/$MAPbI_3$/PTAA/MoO_3/Ag) following steps described below. (i) PCBM solution (20 mg mL^{-1} in CB) was then spin-coated onto the perovskite film at 2000 rpm for 30 s, followed by the spin-coating of a BCP solution (0.5 mg mL^{-1} in ethanol) at 3000 rpm for 30 s. After this, the samples were transfer to the vacuum chamber for the deposition of 100 nm Ag (or Au) under a high vacuum of 5×10^{-4} Pa. (ii) PTAA was deposited from a solution dissolved in 1 mL of toluene contained 15 mg of PTAA, 7.5 µL of LiTFSI stock

solution (520 mg/mL in acetonitrile) and 7.5 μL of tBP. The PTAA solution was spun onto the perovskite film at 4000 rpm for 30 s. Finally, a 10 nm MoO_3 and 100 nm silver (Ag) were thermally evaporated under vacuum, respectively.

The X-ray diffraction patterns were recorded at a scan rate of 10° min^{-1} on Rigaku Ultima IV X-ray diffractometer with Cu Kα radiation (0.15406 nm). The current density-voltage (*J*-*V*) curves of the devices were measured in glovebox with Keithley 2400 measurement source units under 1 sun, AM 1.5 G spectra from a solar simulator (Taiwan, Enlitech), and the light intensity was calibrated with a standard silicon photovoltaic reference cell. The voltage step is 0.02 V, with a delay time of 10 ms per step, the total scanning rate is 0.095 V s^{-1}. For devices thermal stability test, devices were placed on 100 °C hotplate in glovebox. During the aging period, the devices were taken off the hotplate and cooled to room temperature from time to time for PCE decay tracing. Optical microscope images were taken via Nikon LV100 POL equipped with a digital camera.

Mechanics Simulation

Finite Element Method (Abaqus/Standard) is used to simulate the mechanical behaviours of the PVSCs and 3D solid parts are adopted to represent the $MAPbI_3$, interfacial layer and ITO. We use the 'Tie' constraint to connect two separate surfaces of two various parts together, and there is no relative motion between the two surfaces. The mechanical properties of each part, including the elastic modulus and thermal expansion coefficient, are listed in Table S1, and the lengths and widths are set as 1000 nm × 1000 nm, with the thickness of each layer set as Table S1. An 8-node linear element with reduced integration and hourglass control (C3D8R) is chosen. Along the thickness direction, there are at least 4 layers of elements for each part to ensure the calculation accuracy.

The bottom surface of PVSC (the ITO layer in our simulation) is fixed, in other words, six degrees of freedom of the bottom surface (three of displacements and three of rotations) are fixed, to prevent the PVSC from rigid body translation. The other surfaces of the PVSC are free to deformation. A predefined temperature field with

initial magnitude 20℃ is applied, then modified to 120℃, which is consistent with our experiment. Finally, the Tresca equivalent stress field and deformation field (ε_{11}) of $MAPbI_3$ part are exported for further analysis. In our simulation, direction 1 and direction 2 are along the length and width directions respectively, so they reflect the in-plane stress and strain. Due to the symmetry of PVSC structure, the strains along direction 1 are equivalent to their counterparts along direction 2, so we adopt ε_{11} in this work to show the in-plane deformation.

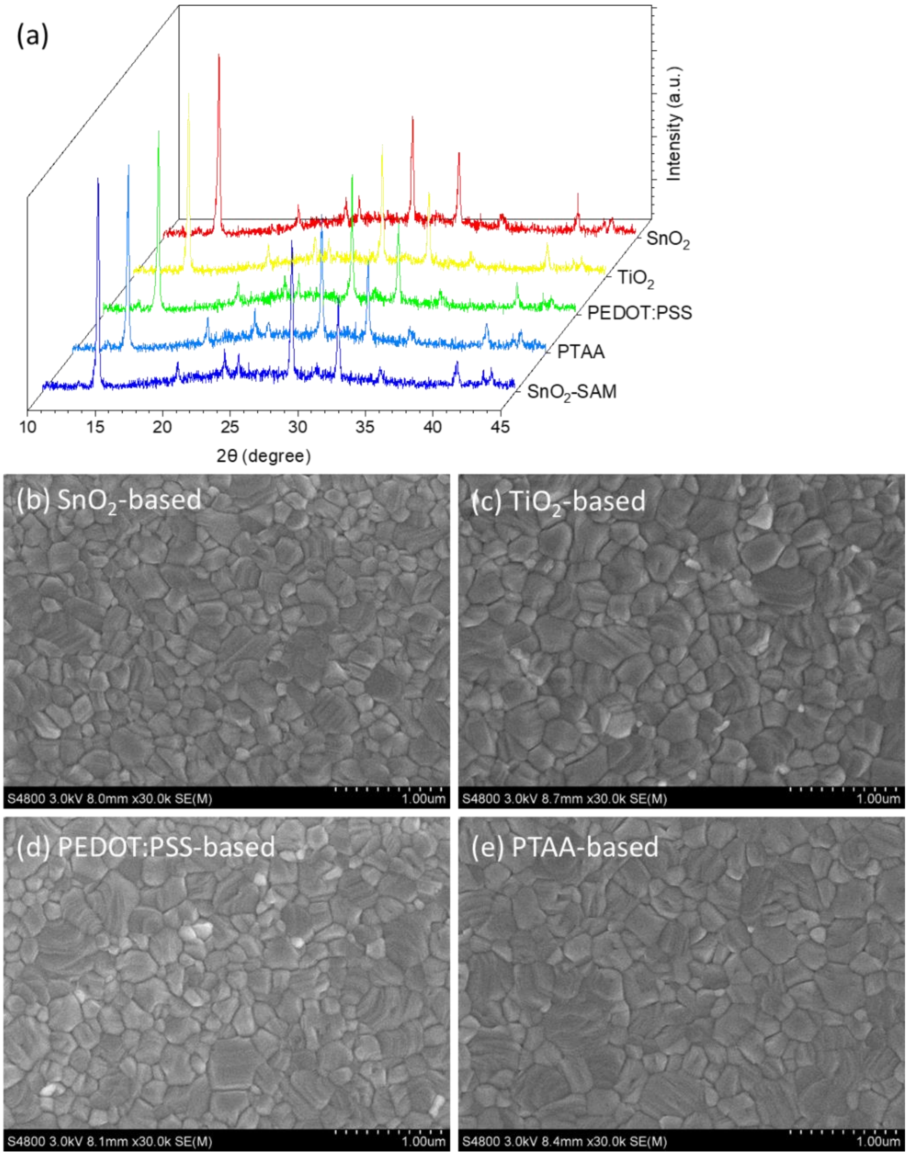

Figure S1. (a) XRD patterns and (b)-(e) SEM images of different perovskite films. Related to Figure 2.

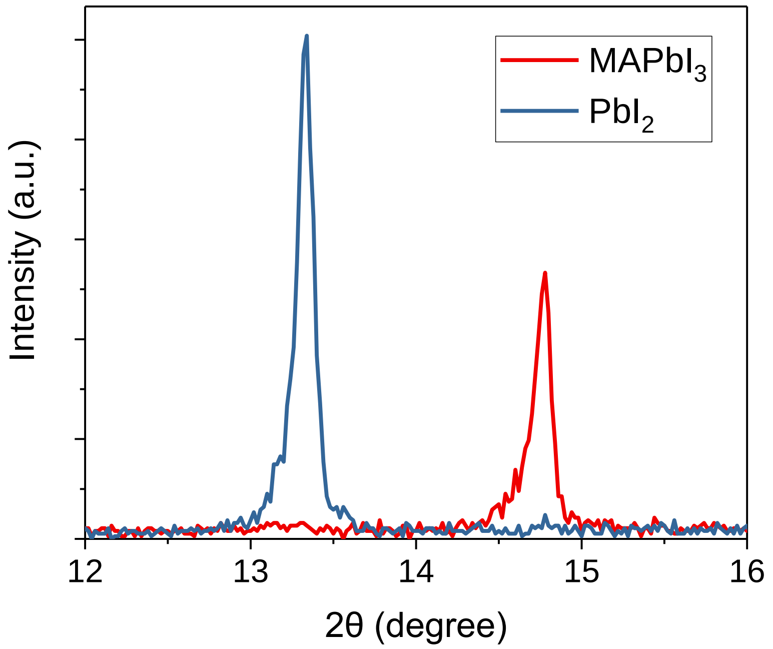

Figure S2. XRD patterns of fresh perovskite film (red line) and totally aged perovskite film (blue line). Related to Figure 2.

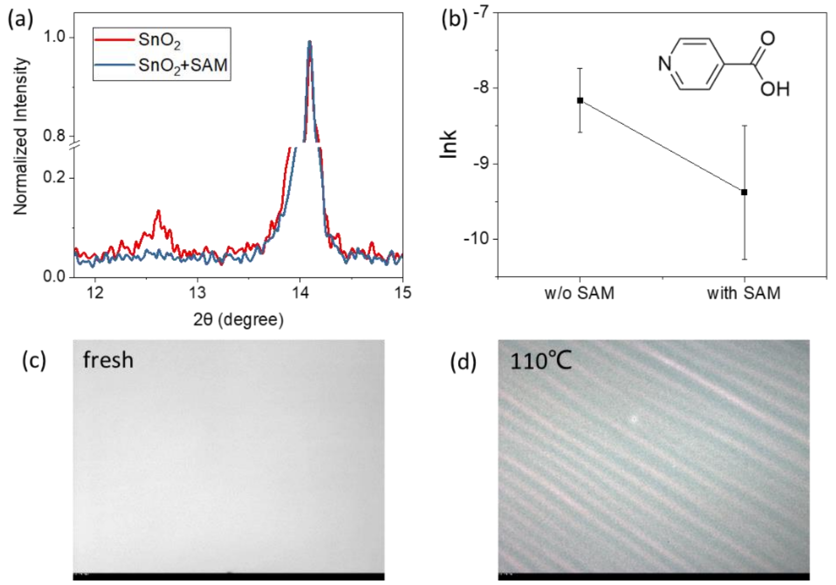

Figure S3. (a) XRD patterns of aged perovskite films based on SnO_2 (red line) and SnO_2-SAM (blue line). (b) lnk of perovskite films on MOs interfacial layers without and with SAM passivation. The inset is the molecular structure of the SAM molecule. Optical microscope images of SnO_2-based perovskite (c) before and (d) after thermal aging at

110 °C. Related to Figure 2.

Table S1. Summary of mechanical parameters involved in the mechanics simulation. Related to Figure 4.

**layers**	**Young's modulus (GPa)**	**Thickness (nm)**
$MAPbI_3$	10~12 (Sun et al., 2015)	300
PEDOT:PSS	1~3 (Lang et al., 2009; Okuzaki et al., 2003)	20
PTAA	~1 (Lee et al., 2019)	5
ITO	118 (Park et al., 2003)	100
SnO_2, TiO_2	>150 (Borgese et al., 2012; Zheng et al., 2011)	20
Metal electrode	6~12	100
PCBM	2 (Awartani et al., 2013)	20

Poisson ratio is set to be 0.3. The thermal expansion coefficient of $MAPbI_3$ is 157×10^{-6} K^{-1}. Due to the small thermal expansion coefficient and large elastic modulus, both the thermal-induced deformation and mechanical-induced deformation of ITO layer are negligible. And the thermal expansion coefficient of other layers is set to be 50×10^{-6} K^{-1}.

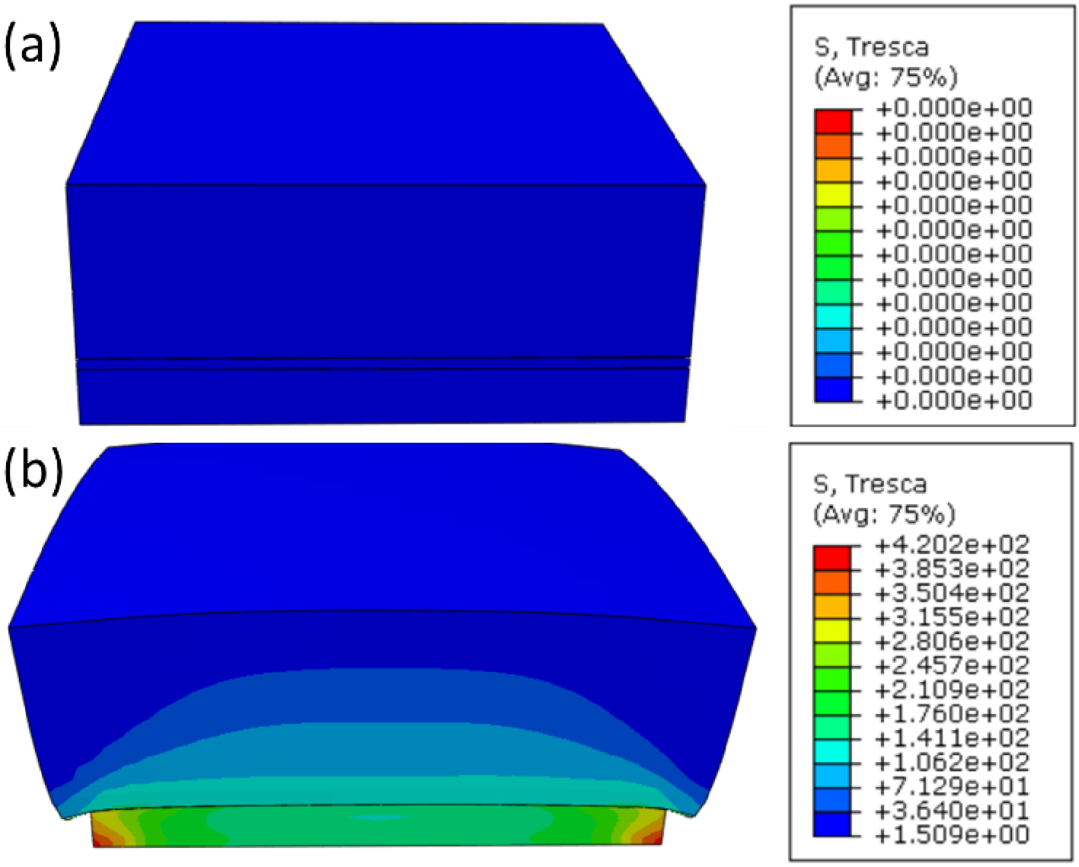

Figure S4. Perovskite film (a) before and (b) after applying temperature field. A deformation scale factor of 10 is used to make the deformation of PVSC obvious. Related to Figure 4.

Because of the constraint effect of substrate on perovskite film, the inner surface of perovskite film is stopped from expansion, whereas the outer surface of perovskite film is rather free to expand. These introduce unbalanced strain within the perovskite film across the thickness direction.

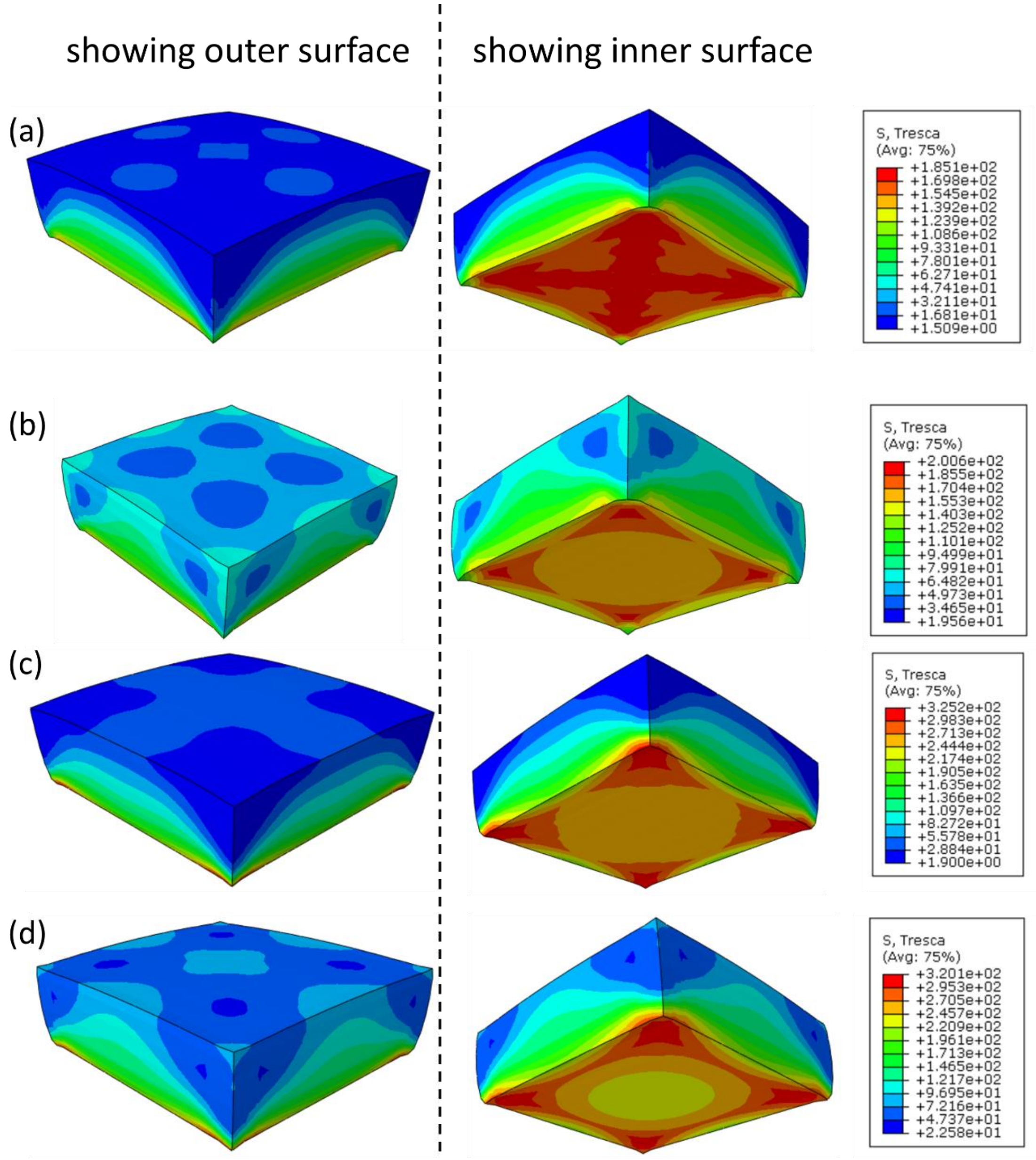

Figure S5. Thermal-induced Tresca stress distribution of perovskite films on interlayer with modulus of 1.5 GPa (a) without and (b) with PCBM layer and counter electrode. Tresca stress distribution of perovskite films on interlayer with modulus of 48 GPa (c) without and (d) with PCBM layer and counter electrode. A deformation scale factor of 10 is used to make the deformation of PVSC obvious. Related to Figure 4.

Herein, increased Tresca stress are observed in both perovskite films without and with counter interfacial layer and electrode when the modulus of interfacial layer increases (from 1.5 GPa to 48 GPa). These results indicate that our conclusion deduced from the simulation based on ITO/interfacial layer/perovskite half device could be extended to that on full device.

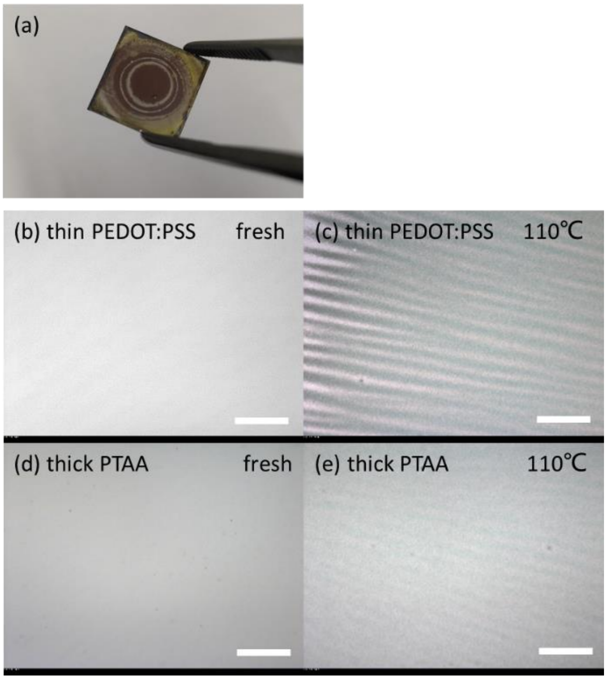

Figure S6. (a) $MAPbI_3$ prepared on thick PTAA based substrate. The thickness of PTAA is ~30 nm. (b)-(e) Optical microscope images of perovskite films deposited on different interfacial layers under thermal aging with different temperatures. Scale-bars in all pictures represent 200 µm. Related to Figure 4.

Table S2. Photovoltaic parameters of PVSCs based on different interfacial layers under the illumination of AM 1.5 G, 100 mW/cm^2. The average values are calculated from 8 devices. Related to Figure 5.

**Interfacial layer**	**V_{OC} (V)**	**J_{SC} (mA/cm^2)**	**FF**	**PCE (%)**
SnO_2	1.07	21.56	0.66	15.26 (14.73±0.48)
TiO_2	1.07	22.25	0.57	13.55 (12.24±1.13)
PEDOT:PSS	1.01	19.33	0.70	13.56 (12.90±0.68)
PTAA	1.07	22.22	0.75	17.83 (16.74±0.91)
TPL	1.08	22.46	0.76	18.78 (17.18±1.00)

Table S3. Optimization of the TPL by varying the thickness of PEDOT:PSS and PTAA, respectively. Photovoltaic parameters of PVSCs are taken under the illumination of AM 1.5 G, 100 mW/cm^2. The average values are calculated from 8 devices. Related to Figure 5.

**Thickness (nm)**		**V_{OC} (V)**	**J_{SC} (mA/cm^2)**	**FF**	**PCE (%)**
PEDOT:PSS	PTAA				
24	7	1.02	18.13	0.76	14.03 (13.94±0.09)
	3	1.02	18.33	0.78	14.68 (13.85±0.68)
14	7	1.08	22.46	0.76	18.78 (17.18±1.00)
	3	0.97	19.79	0.74	14.39 (13.87±0.49)
12	7	1.07	21.73	0.74	17.49 (16.94±0.52)
	3	0.98	19.78	0.74	14.38 (13.63±0.47)

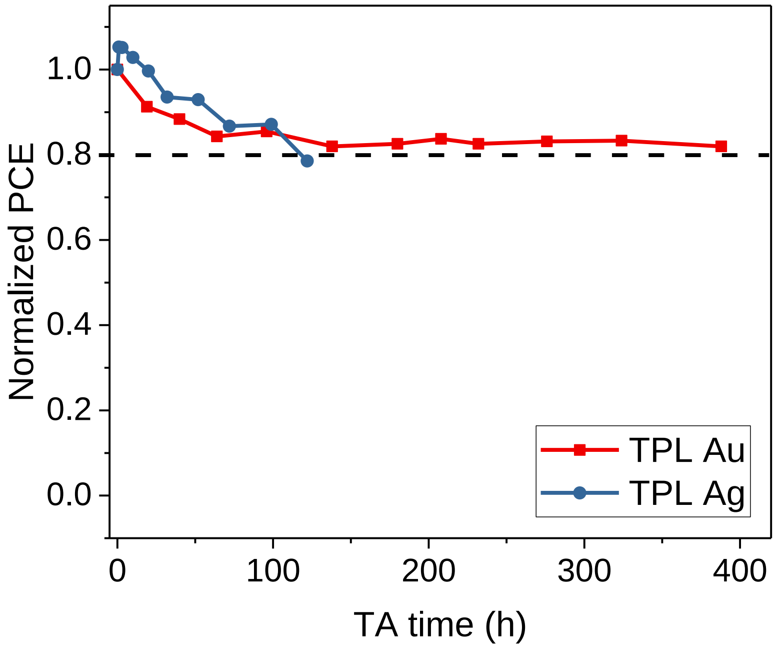

Figure S7. PCE evolution of PVSCs based on TPL with Ag (blue line) and Au (red line) electrode heated at 100°C in glovebox. Related to Figure 5.

References

Zhou, H., Chen, Q., Li, G., Luo, S., Song, T.-b., Duan, H.-S., Hong, Z., You, J., Liu, Y., and Yang, Y. (2014). Interface engineering of highly efficient perovskite solar cells. Science *345*, 542-546.

Zuo, L., Chen, Q., De Marco, N., Hsieh, Y.-T., Chen, H., Sun, P., Chang, S.-Y., Zhao, H., Dong, S., and Yang, Y. (2017). Tailoring the interfacial chemical interaction for high-efficiency perovskite solar cells. Nano Lett. *17*, 269-275.

Sun, S., Fang, Y., Kieslich, G., White, T. J., and Cheetham, A. K. (2015). Mechanical properties of organic–inorganic halide perovskites, $CH_3NH_3PbX_3$ (X = I, Br and Cl), by nanoindentation. J. Mater. Chem. A *3*, 18450-18455.

Lang, U., Naujoks, N., and Dual, J. (2009). Mechanical characterization of PEDOT:PSS thin films. Synth. Met. *159*, 473-479.

Okuzaki, H., and Ishihara, M. (2003). Spinning and characterization of conducting microfibers. Macromol. Rapid Commun. *24*, 261-264.

Lee, I., Rolston, N., Brunner, P.-L., and Dauskardt, R. H. (2019). Hole-transport layer molecular weight and doping effects on perovskite solar cell efficiency and mechanical behavior. ACS Appl. Mater. Interfaces *11*, 23757-23764.

Park, S. K., Han, J. I., Moon, D. G., and Kim, W. K. (2003). Mechanical stability of externally deformed indium–tin–oxide films on polymer substrates. Jpn. J. Appl. Phys. *42*, 623-629.

Borgese, L., Gelfi, M., Bontempi, E., Goudeau, P., Geandier, G., Thiaudière, D., and Depero, L. E. (2012). Young modulus and Poisson ratio measurements of TiO_2 thin films deposited with Atomic Layer Deposition. Surf. Coat. Technol. *206*, 2459-2463.

Zheng, Y., and Geer, R. E. (2011). Namomechanical imaging and nanoscale elastic modulus measurements of SnO_2 nanobelts. MRS Proceedings *821*, P2.3.

Awartani, O., Lemanski, B. I., Ro, H. W., Richter, L. J., DeLongchamp, D. M., and O'Connor, B. T. (2013). Correlating stiffness, ductility, and morphology of polymer:fullerene films for solar cell applications. Adv. Energy Mater. *3*, 399-406.
